# Cultural Components of Sex Differences in Color Preference

**DOI:** 10.1111/cdev.13528

**Published:** 2021-01-21

**Authors:** Jac T. M. Davis, Ellen Robertson, Sheina Lew‐Levy, Karri Neldner, Rohan Kapitany, Mark Nielsen, Melissa Hines

**Affiliations:** ^1^ University of Cambridge; ^2^ Vrije Universiteit Amsterdam; ^3^ Simon Fraser University; ^4^ Aarhus University; ^5^ University of Queensland; ^6^ Keele University; ^7^ University of Johannesburg

## Abstract

Preferences for pink and blue were tested in children aged 4–11 years in three small‐scale societies: Shipibo villages in the Peruvian Amazon, kastom villages in the highlands of Tanna Island, Vanuatu, and BaYaka foragers in the northern Republic of Congo; and compared to children from an Australian global city (total *N* = 232). No sex differences were found in preference for pink in any of the three societies not influenced by global culture (*d*s − 0.31–0.23), in contrast to a female preference for pink in the global city (*d* = 1.24). Results suggest that the pairing of female and pink is a cultural phenomenon and is not driven by an essential preference for pink in girls.

1

The color pink is widely used as a marker for female gender, in mass communication, mass media, and mass‐produced children’s clothes and toys (Auster & Mansbach, 2012; Koller, 2008; Merler, Cao, & Smith, 2015; Sweet, 2013; Vaisman, 2016). Debate over the appropriate use of color, especially pink, to signal gender has become increasingly polarized in academic fields (Del Giudice, 2012, 2017; Fine & Rush, 2018; Liben & Bigler, 2002; Sweet, 2013), in government (White House Office of the Press Secretary, 2016), and in the popular press (Gonchar, 2015). At the root of this debate is the question: is culture influencing children’s color preferences, or do girls innately prefer pink?

The use of pink as a marker for female gender has spread rapidly across global consumer and media culture (Sweet, 2013). Contemporary global information flows, such as mass media, mass communications, and mass‐produced goods, encourage the transmission of transnational cultural ideas such as the pairing of pink and female (Featherstone, 1990). On the internet, pink is used to signal content for girls in online toy stores (Auster & Mansbach, 2012), on magazine websites (Koller, 2008), on blogs (Vaisman, 2016), and in social networks (Fortmann‐Roe, 2013), and pink is used as a marker for female gender on television (Kolbe & Muehling, 1995). Perhaps as a result of these media and communications, parents are likely to buy pink toys, clothes, and furniture for girls and avoid them for boys (Fisher‐Thompson, 1993; Jonauskaite et al., 2019; Pomerleau, Bolduc, Malcuit, & Cossette, 1990). Due to the rapid expansion of mass communication and mass media, messages about pink as a marker of female gender are more widely available than ever (Sweet, 2013).

Children’s preferences and behaviors appear to support the cultural pairing of pink and female. Research on children’s color preferences consistently finds a female preference for pink in industrialized countries such as the United States (LoBue & DeLoache, 2011; Weisgram, Fulcher, & Dinella, 2014), the United Kingdom (Wong & Hines, 2015), Hong Kong (Yeung & Wong, 2018), Iran (Mohebbi, 2014), Switzerland (Jonauskaite et al., 2019; Zentner, 2001), and Canada (Chiu et al., 2006). The female preference for pink is seen consistently, whether color preference is measured using laminated colored squares (Chiu et al., 2006; Wong & Hines, 2015; Yeung & Wong, 2018), cardboard rectangles (Zentner, 2001), online color swatches (Jonauskaite et al., 2019), or pairs of toys with one toy colored pink (LoBue & DeLoache, 2011; Weisgram et al., 2014). The current research consensus is therefore that, on average, girls consistently prefer pink while boys avoid it.

The consistent sex difference in preference for pink has prompted theory development about essential differences between boys and girls that might produce universal sex differences in color preferences (Alexander, 2003; Del Giudice, 2017; Franklin, Bevis, Ling, & Hurlbert, 2010; Hurlbert & Ling, 2007; Hurlbert & Owen, 2015). Biological processes, in particular, have been proposed as essentialist explanations for sex differences in human color preferences (Alexander, 2003). These essentialist explanations tend to focus on sex differences in preferences for specific colors, and in particular on a female preference for reddish‐pink hues (Del Giudice, 2017). Thus, essentialist theories of color preference tend to focus on female preferences for pink as a key marker of universal sex differences in color preferences.

One essentialist theory, cone‐contrast theory (Hurlbert & Ling, 2007), posits that color preferences are a biological adaptation based on evolved characteristics of the human visual system (Hurvich & Jameson, 1957). Retinal cone photoreceptors are sensitive to different, but overlapping, wavelengths of colors, short (bluish colors), medium (greenish colors), or long (reddish colors). The neural signals received by different photoreceptors can be contrasted to give a measurement of color processing, approximated by comparing long‐wavelength and medium‐wavelength cone signals (the L‐M opponent process) and then by comparing short‐wavelength signals with the combined long‐ and medium‐wavelength cone signals (the S − (L + M) opponent process) (De Valois & De Valois, 1993). Individual differences in these neural dimensions of color processing are thought to lead to individual differences in color preferences.

Individual differences in neural color processing have been extended to explain sex differences in color preferences (Hurlbert & Ling, 2007; Hurlbert & Owen, 2015). One study asked British and Chinese adults, all of whom were living in a large British city (Hurlbert & Ling, 2007), to select their preferred color from a series of pairs of rectangles, and then analyzed the preference responses with color curve analyses. Color preferences were best explained by two components that corresponded to short‐axis (S‐axis) and long‐medium‐axis (L‐M axis) neuronal cone‐opponent contrast channels. The analysis found sex differences in participants’ preferences for the L‐M axis, which runs approximately from reddish to blue‐green hues. Female participants tended to prefer reddish‐purple hues, independently of brightness and saturation, while male participants tended to prefer blue‐green hues, although to a lesser extent. The authors hypothesized that women’s preference for pink might be linked to neuronal receptors for the long‐medium‐axis (L‐M axis) in the female brain, and argued for an essential difference between male and female visual‐neural color processing.

The cone‐contrast approach has been tested directly, and studies find no sex difference in the L‐M component for predicting color preferences in infants (Franklin et al., 2010), or in Japanese or US adults (Yokosawa, Schloss, Asano, & Palmer, 2016). These results suggest that the L‐M differences observed in Hurlbert and Ling (2007) are likely to depend on culture, rather than biology. Recent reanalysis of the cone‐contrast approach suggests that it may be useful for describing and predicting patterns of color preferences, but that it may have serious limitations for explaining the origin of color preferences (Schloss, Lessard, Racey, & Hurlbert, 2018). However, while the cone‐contrast approach itself may be increasingly critiqued, the general idea that a preference for pink is an essential part of female evolutionary history persists (Del Giudice, 2017).

Proponents of essentialist theories also suggest that women’s preference for pink might be a biological adaptation to female‐specific environmental cues that were important in human evolutionary history, such as foraging for red fruits, or selecting healthy male mates (Alexander, 2003; Hurlbert & Owen, 2015). Red hues are hypothesized to facilitate the identification of edible fruits and leaves by female “gatherers,” who would need to be more aware of color than male “hunters” (Hurlbert & Ling, 2007). Alternatively, subtle changes in primates’ skin color due to sexual states are hypothesized to link with women’s evolutionary role as caregivers (Hurlbert & Ling, 2007), or to signal masculinity, with male faces being more reddish in hue than female faces (Alexander, 2003).

The evidence for an evolutionary function of pink, however, is tenuous. If pink were linked to foraging, then one would expect to see a female preference for pink in non‐human foraging primates that are evolutionarily close to humans. Yet, a study of adult gorillas and chimpanzees found no sex differences in their preferences for blue, green, or red stimuli (Wells, McDonald, & Ringland, 2008). Furthermore, if pink were a marker of healthy male mates, then homosexual men should like pink as much as heterosexual women do. However, a study of North American college students found that non‐heterosexual men showed similar color preferences to heterosexual men, and did not like pink as much as heterosexual women did (Ellis & Ficek, 2001). Finally, if pink were a marker of healthy male mates, then adult women should show a larger preference for pink than young girls do, because mate information would be more relevant after women reach reproductive age. But several studies find that adult women prefer pink or reddish hues, less than young girls do (Jonauskaite et al., 2019; Ling & Hurlbert, 2011). Furthermore, although adult women like pink more than men do, they may not prefer pink to blue (Wong & Hines, 2015).

In contrast to the essentialist view, others argue that children’s color preferences are influenced by social and cultural messages about pink as a marker for female gender (Fine & Rush, 2018; LoBue & DeLoache, 2011; Pomerleau et al., 1990; Sweet, 2013; Wong & Hines, 2015). One such non‐essentialist theory is schema theory. Social messages, such as parents buying gendered toys (Pomerleau et al., 1990), or cultural messages, such as gendered toy advertising (Kolbe & Muehling, 1995; Pastor, Nicolás, & Salas, 2013), may influence children’s construction of gender schema: large associative networks of information about gender that children assemble based on their experiences and observations of the world (Bem, 1981; Liben & Bigler, 2002; Martin & Halverson, 1981). Because gender is a functionally significant dimension in society, it is salient to children, who notice the associations between gender and environmental features such as color (Bigler & Liben, 2007). Children actively search for information about what is associated with their gender (Martin & Ruble, 2004), and cognitively assimilate this information, such as the link between pink and female, into their gender schemas. Children then adjust their behavior to match what they consider appropriate for their gender, based on their gender schemas (Carter & Levy, 1988; Liben & Bigler, 2002; Martin & Halverson, 1981).

Another non‐essentialist theory, ecological valence theory (Palmer & Schloss, 2010), holds that humans develop preferences for colors that are associated with pleasant experiences. As an adaptive strategy, humans use color as a heuristic signal of good or bad objects or environments, and therefore people prefer colors that are associated with objects that characteristically have advantageous functions for survival, reproductive success, or social cohesion (Palmer & Schloss, 2010). The ecological valence theory is agnostic about whether sex differences in color preferences are essential (Taylor, Schloss, Palmer, & Franklin, 2013), but it does predict that color preferences may change with social context, especially for colors with strong ties to social institutions, such as gender (Palmer & Schloss, 2010).

Researchers looking to identify which aspects of color preference might be innate, and which might be learned, have turned to research on infants. If sex differences in color preference were linked to experience, one would expect to see no sex differences in infants’ color preferences, and experience‐dependent sex differences in color preferences in older children and adults. Indeed, research has found that infants aged 3–24 months show no sex differences in color preferences (Franklin et al., 2010; Jadva, Hines, & Golombok, 2010; Zemach, Chang, & Teller, 2007), that girls’ preference for pink increases between ages 2 and 5 years (LoBue & DeLoache, 2011; Wong & Hines, 2015), and that adults’ experiences with colored objects are statistically linked to their color preferences and to the emotional valence of the objects (Palmer & Schloss, 2010). However, infants do show color preferences unrelated to gender (Franklin et al., 2010; Taylor, Schloss, et al., 2013; Zemach et al., 2007), and infants old enough to be tested for color preferences may have already had pleasant or unpleasant experiences with colors (Taylor, Schloss, et al., 2013). Overall, the existing evidence from infant research generally supports a learning account of sex differences in color preferences, but does not rule out a role for essential, adaptive functions of color preferences in general.

Together, this evidence suggests that color preference is part of the social construction and experience of gender that comes with culture, including global culture. Messages about pink as a marker of female gender could be delivered through mass media (Kolbe & Muehling, 1995), mass communication (Auster & Mansbach, 2012; Vaisman, 2016), or mass‐produced children’s products (Pomerleau et al., 1990; Sweet, 2013), and could therefore influence children in any industrialized or semi‐industrialized context with access to these aspects of global culture. But to determine whether girls’ preference for pink is influenced by mass media, mass communication, or mass‐produced children’s products, we would need to see whether girls prefer pink more than boys do in settings without access to these aspects of global culture.

To date, no published research has tested children’s color preferences in settings without access to the above aspects of global culture, but some research of this kind has been done in adults. No female preference for pink was found in semi‐nomadic Himba adults in northern Namibia (Taylor, Clifford, Clifford, & Franklin, 2013), or in Hadza hunter‐gatherer adults in Tanzania (Groyecka, Witzel, Butovskaya, & Sorokowski, 2019), but in two studies, adult women in a remote Yali village in Papua New Guinea were more likely to select reddish colors than men were (Groyecka et al., 2019; Sorokowski, Sorokowska, & Witzel, 2014). Finally, a female preference for pink has been demonstrated in adults from India (Bonnardel, Beniwal, Dubey, Pande, & Bimler, 2018), Saudi Arabia (Al‐Rasheed, 2015), and China (Hurlbert & Ling, 2007; Ou, Luo, Woodcock, & Wright, 2004; Yeung & Wong, 2018), but these countries are industrialized and likely to have gender schema and experiences that are influenced by global culture. Taken together, these results are inconclusive about the effects of global culture on sex differences in adults’ color preferences, and they give no hints about whether global culture might affect children’s preference for pink.

Scholars disagree on how to interpret the existing research and what it means for theories on the origins of sex differences in color preferences. Some recent papers have interpreted the existing cross‐cultural research as supporting essentialist, biological origins of sex differences in color preferences (e.g., Bonnardel et al., 2018; Del Giudice, 2017), while others have rejected this interpretation in favor of a non‐essentialist, cultural perspective (e.g., Al‐Rasheed, 2015; Groyecka et al., 2019) and called for studies that explicitly test the influence of culture versus biology (Jonauskaite et al., 2019). The effects of global culture on children’s gendered preferences, including color preferences, therefore remain a topic of scholarly debate.

The rapid expansion of mass media, mass communication, and mass‐produced goods across the globe makes it timely to assess the influence of global culture on children’s gendered preferences and behaviors, including children’s preference for pink. The research presented here was thus aimed at charting children’s preference for pink in settings with limited exposure to global culture. If biological essentialist theories, such as cone‐contrast theory, were true, we would expect to see a female preference for pink in settings with limited exposure to global culture. Alternatively, if non‐essentialist theories, such as schema theory and ecological valence theory, were true, then we would expect to see no gender difference in children’s preference for pink in these settings.

In the present study, we investigate the preference for pink and blue in different cultures. Pink is a useful litmus test for gender‐typed preferences, as it is used consistently as a marker for female gender in global culture, and boys and girls in industrialized nations consistently show sex differences in their preferences for pink (Pomerleau et al., 1990). Furthermore, red‐blue contrasts, including pink, are the most important for finding sex differences according to cone‐contrast theory (Hurlbert & Owen, 2015). Pink has also been singled out in non‐essentialist theories of gender (Weisgram et al., 2014), and prior studies of children’s sex‐typed color preferences have focused on red and blue hues (Jadva et al., 2010; Wong & Hines, 2015). Thus, testing sex differences in children’s preference for pink in small‐scale societies may reveal cultural components of sex differences in color preference.

## 2 Method

### 2.1 Participants

**Figure 1:**
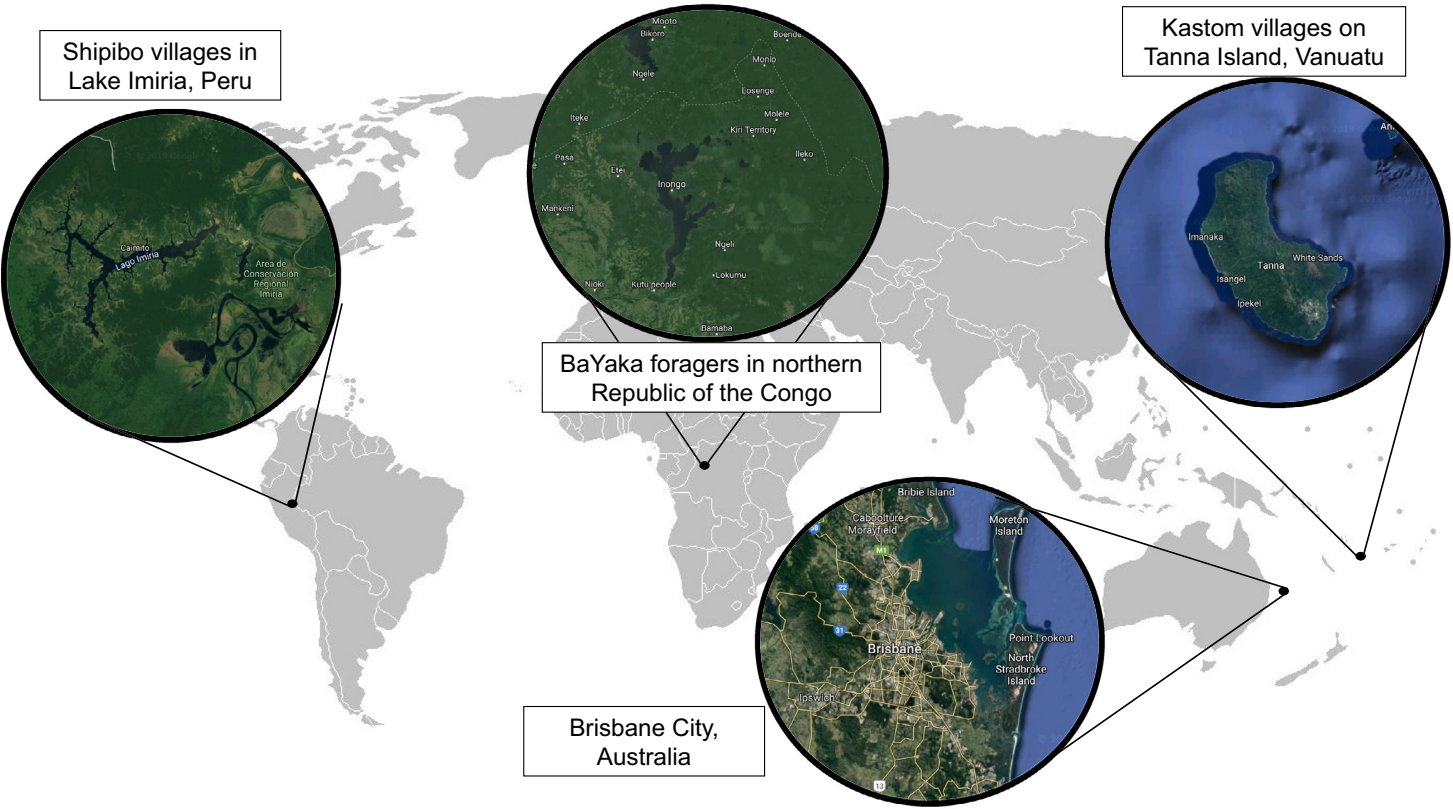
Study site locations and satellite maps to show land cover and urban density. Areas with lower urban density are assumed to have less contact with global cultural norms, including the pairing of pink with female gender.

Participants were recruited from four populations: children living in remote villages in the Shipibo communities of lowland Peru; children living in remote villages in the *kastom* Navhal‐speaking communities of Tanna Island, Vanuatu; children in hunter‐gatherer BaYaka communities in Northern Congo; and, for comparison, children in a global city in Australia (see Figure 1).

Study regions were sought that had low probability of contact with global cultural norms, including the pairing of pink with female gender. As a proxy for contact with global cultural norms, we selected areas with low urban density, high distance to urban centers, and where the closest urban center had low population and built‐up area according to the Global Human Settlement project (Florczyk et al., 2019). These indicators are reported in Table 1. Additionally, we gathered qualitative information on each site’s cultural flows: mass media, mass communications, and mass‐produced toys; and on gender roles in each site. This qualitative information is reported below. In each site, participants were children between the ages of 4 and 11 years. Participant characteristics are reported in Table 2.

**Table 1:** Study Samples and Nearest Urban Center (NUC) Characteristics

Study sample	Population group	Country	NUC	Distance to NUC (km)	Population of NUC	NUC built‐up area (km^2^)
Lake Imiria	Shipibo	Peru	Pucallpa	56.06	341,919	28.1
Tanna Island	Kastom	Vanuatu	Port Vila	225.39	51,437	5.37
Northern Congo	BaYaka	Republic of Congo	Impfondo	293.7	179,462	14.13
Brisbane City	—	Australia	—	0	990,070	303.6

**Table 2:** Participant Characteristics for Each Site. Age is Approximate for Tanna and BaYaka Participants

Participant group	Number	Age in years (*SD*)
Shipibo, Peru		
M	45	6.91 (1.77)
F	54	6.65 (1.86)
Tanna, Vanuatu		
M	31	6.32 (2.01)
F	19	6.32 (1.89)
BaYaka, Congo		
M	33	7.92 (2.11)
F	18	8.73 (2.70)
Brisbane City, Australia		
M	30	5.78 (1.87)
F	20	5.67 (1.62)
Total		
M	139	6.78 (2.05)
F	111	6.75 (2.18)

### 2.2 Site Characteristics

#### 2.2.1 Shipibo Villages in Lake Imiria Region, Peru

##### 2.2.1.1 Participants

Participants in Peru were 100 children (45 boys and 55 girls). All participants were part of the Shipibo indigenous group and the task was delivered in Spanish or Shipibo language, depending on the translator’s assessment of which language the child understood best.

##### 2.2.1.2 Places in study

Data were collected in 2015. At the time of data collection, Caimito, Buenos Aires, Nuevo Loreto, and Nueva Yarina were four villages of indigenous Shipibo people, situated in the Lake Imiria region of the Ucayali River in the Amazon rainforest, Peru. Villagers lived primarily on small‐scale agricultural and fishing subsistence, but each village contained a small number of people who were paid a government wage for teaching, tax collecting, or other professions. Peruvian government and aid agencies occasionally visited Caimito village.

##### 2.2.1.3 Education and language

Children attended state‐regulated formal education. Lessons were taught in Shipibo language and in Spanish. Adults and children spoke Shipibo and some adults also spoke Spanish.

##### 2.2.1.4 Nearest urban center

The closest urban center was Pucallpa, a small town approximately 56 km away. The Lake Imiria region had no road access from Pucallpa and was reachable only by a combination of foot and boat travel. Travel between villages was also on foot through the jungle, or by boat.

##### 2.2.1.5 Mass media and mass communications

The villages had no central electricity or communications infrastructure, but they did typically have a generator that supplied electricity to the town when fuel was available. Media was morning broadcasts of news over a loudspeaker in the village center. Telecommunications were provided by a single landline phone in each village center. Caimito village had mobile reception, although the mobile tower was set up only a few months before our visit and almost no residents owned a mobile phone. There was no Internet access.

##### 2.2.1.6 Mass‐produced items

Each village contained a small shop selling some mass‐produced food and household items, but no toy or leisure items. Adults and children wore mass‐produced clothes, typically sourced second hand or provided by charities. Some of these clothes, especially clothes for children, depicted media characters or children’s mass‐produced toys.

##### 2.2.1.7 Gender in culture

Shipibo culture is patrilineal, and roles were gender differentiated (e.g., only men could fish in the river). Village chiefs and administrators were not exclusively men in theory, but in practice, during this research, only men were observed in these roles. Women had primary responsibility for child care, starting from a young age, and girls were expected to help care for younger siblings.

##### 2.2.1.8 Recruitment

Children in the Shipibo villages were recruited through a formal process. The research project was presented to the village in a public forum, where anyone could ask questions about the project or about the researchers. In the three smaller villages (Buenos Aires, Nueva Yarina, and Nuevo Loreto), all children who fell within the required age range were recruited. In the larger village (Caimito), children were selected for the study via a process suggested and overseen by the village leaders. Mothers nominated their children for participation in a village meeting. Children were given a small gift for participating (some books and pencils for school).

#### 2.2.2 *Kastom* Villages on Tanna Island, Vanuatu

##### 2.2.2.1 Participants

Participants in Vanuatu were 51 children (31 boys and 20 girls). All participants were part of the Navhal language group and the task was delivered in both Navhal and Bislama languages, depending on the translator’s assessment of which language the child understood best.

##### 2.2.2.2 Places in study

Data were collected in 2016. At the time of data collection, Ikunala and Yakel were *kastom* villages in the remote mountain regions of Tanna Island, Vanuatu. Both villages lived according to *kastom* tradition. *Kastom* tradition limited contact with modern inventions: villagers wore skirts and penis sheaths made of grass, lived in grass houses, and were encouraged to avoid modern influences (Lindstrom, 2008). In practice, we observed villagers using some clothes, blankets, and cooking equipment that must have been purchased outside the *kastom* villages. The *kastom* villages were located in the highlands in central Tanna Island. Ikunala and Yakel were the lowest of the villages and were accessible via dirt road in dry weather. Additional participants traveled down from smaller villages that were higher up in the hills and only accessible on foot. Ikunala village was not typically accessible to outsiders, but Yakel village allowed access from paying visitors including tourists and film crews.

##### 2.2.2.3 Education and language

Children did not receive a formal education and did not typically travel to large towns or cities, although adults sometimes visited Lenakel to take produce to market. Adults and children spoke the indigenous Navhal language and some adults also spoke Bislama.

##### 2.2.2.4 Nearest urban center

The closest town was Lenakel, the capital of Tanna Island. At the time of data collection, Vanuatu had no urban centers according to global definitions (Florczyk et al., 2019) and the distance from Tanna to Port Vila, the capital of Vanuatu, was approximately 225km. Port Vila was accessible from Tanna via air or boat travel between the islands. Travel on the island was typically by foot, but vehicles occasionally visited the *kastom* villages when the road was dry.

##### 2.2.2.5 Mass media and mass communications

The *kastom* villages had no radio, television, or other media access. There was no infrastructure for electricity and no electrical devices or generators, but some villagers had small solar‐powered torches. The villages had no mobile coverage. There was no Internet. However, some villagers owned mobile phones.

##### 2.2.2.6 Mass‐produced items

*Kastom* villages had no shops but had some inter‐village trade of valuable goods such as woven mats and baskets. Money was rarely used, and having money was not seen as culturally desirable among village members.

##### 2.2.2.7 Gender in culture

The *kastom* culture is patrilocal, patrilineal, and monogamous (Lindstrom, 2008). Women could own land and livestock, but positions of power in the village (chief, medicine man, spiritual leader) were always held by men. Women had primary child care responsibilities.

##### 2.2.2.8 Recruitment

In each village, two translators were recruited who each spoke Navhal, Bislama, and English, and who knew children of the village personally. According to translators’ advice, and after consulting the chiefs, we provided each village with appropriate gifts to thank them for their participation: Ikunala village with trade gifts (coconuts, tinned food, rice, and kava, approximately 70 GBP [British Pound Sterling] worth), and Yakel village with money (10,000 vatu, equivalent in value to the gift for Ikunala village). Children were recruited through village chiefs and heads of families. Since kastom children did not know their age in years, age was estimated by a knowledgeable adult from the community. All children in each village who appeared to be in the target age group, and who were otherwise available and eligible to participate, were included in our study sample.

#### 2.2.3 BaYaka Village in the Congo Basin, Republic of the Congo

##### 2.2.3.1 Participants

Participants in Congo were 51 children (18 girls and 33 boys). Tasks were delivered in the BaYaka (di.Aka) language.

##### 2.2.3.2 Places in study

Data were collected in 2018 in a remote multi‐ethnic village of approximately 400 inhabitants in the Likouala region of Northern Congo‐Brazzaville. At the time of data collection, the village was accessible only on foot or by boat. BaYaka primarily relied on forest products for subsistence, including hunted meat, white tubers, honey, fruit, caterpillars, and fish. Many BaYaka also planted low‐maintenance gardens of cassava and bananas. BaYaka spent approximately 6 months of the year in forest camps, and 6 months of the year in a village setting.

##### 2.2.3.3 Education and language

While the BaYaka had access to schools, children infrequently attended, and schools were often closed during the school year. Instead, children spent much of their time in the multi‐aged, mixed‐sex playgroup, where most basic subsistence skills were acquired through play, participation and teaching in early and middle childhood (Lew‐Levy, Boyette, Boyette, Crittenden, Hewlett, & Lamb, 2019; Lew‐Levy, Crittenden, et al., 2019; Lew‐Levy et al., 2020). Adults and parents spoke BaYaka to each other, and many also spoke Lingala, the local trade language.

##### 2.2.3.4 Nearest urban center

The closest urban center was Impfondo, a small town almost 300km away. At the time of data collection, participants rarely traveled to Impfondo. Thanry, a logging town, was approximately 40 km away by foot from the village. BaYaka sometimes traveled to Thanry to seek employment at the logging company, visit with kin, or trade forest goods for market goods.

##### 2.2.3.5 Mass media and mass communications

No running water, electricity, or cell phone reception was available.

##### 2.2.3.6 Mass‐produced items

The village had two small stores from which market goods (e.g., flashlights, fishing hooks, rice) could be purchased. The BaYaka maintained trade relationships with their farmer neighbors, with whom they exchanged forest goods such as hunted meat or honey for market goods.

##### 2.2.3.7 Gender in culture

BaYaka were bilocal and most marriages were monogamous. BaYaka maintained relatively egalitarian gender relationships (Lewis, 2017), and fathers played a central role in child caretaking (Boyette, Lew‐Levy, Sarma, Valchy, & Gettler, 2019).

##### 2.2.3.8 Recruitment

Community consent was obtained during a village meeting prior to the start of data collection. Researchers then conducted a census of all houses. Using this census, a list of children within the desired age range was generated. Since the BaYaka do not know their age in years, we estimated ages following the procedures outlined by Diekmann et al. (2017). Consent was obtained from all parents of children eligible to participate, and immediately prior to the experiment, child assent was also obtained. Children were given a candy at the end of the experiment, and all participating households received a mosquito net, a knife, and a serving spoon as a gift at the end of the field season.

#### 2.2.4 Brisbane City, Australia

##### 2.2.4.1 Participants

Participants in the City sample were 50 children (30 boys and 20 girls). Children were predominantly white and had English as a first language. The task was conducted in English.

##### 2.2.4.2 Places in study

Data were collected in 2016. At the time of data collection, Brisbane was typical of an industrialized city usually studied in academic research on color preference. It was a large city (population approx. 2.35 million) with links to the global economy.

##### 2.2.4.3 Education and language

Brisbane was an English‐speaking city with compulsory free education for children aged 5 years and older.

##### 2.2.4.4 Nearest urban center

Brisbane was an urban center and a state capital in Australia.

##### 2.2.4.5 Mass media and mass communications

Brisbane had large‐scale energy infrastructure and telecommunications infrastructure, and most people had access to mass media through radio, television, and Internet. Mobile coverage was provided by multiple carriers for phone calls and data. Most people in Brisbane had access to a mobile phone including Internet.

##### 2.2.4.6 Mass‐produced items

Mass‐produced toys were readily available through large international chain stores, local toy stores, and online.

##### 2.2.4.7 Gender in culture

Gender roles were typical of large‐scale industrialized societies of the type commonly studied in color preference research.

##### 2.2.4.8 Recruitment

Children in Brisbane were recruited as a convenience sample in a public, free‐entry museum centered in the middle of the city and well attended by a large cross‐section of the population. Consent was given verbally and electronically by parents, and children were given a wristband for participating.

### 2.3 Materials and Procedure

Stimuli were presented as printed, laminated pages, following the most common procedure for assessing color preference in children (Chiu et al., 2006; Wong & Hines, 2015; Yeung & Wong, 2018; Zentner, 2001). Children were shown one page at a time. Each page depicted two colored options. Children were shown each pair of stimuli and asked to point to the option that they preferred.

Five pairs of stimuli were used to assess children’s preference for pink. Three pairs of stimuli compared pink (hue = 234, saturation = 235, luminance = 191) and blue (hue = 146, saturation = 240, luminance = 115). To examine preferences for pinkish/reddish colors and bluish colors more broadly, two additional color pairs were created: red (hue = 234, saturation = 235, luminance = 115), versus the original blue and the original pink versus pale blue (hue = 146, saturation = 240, luminance = 191). These stimuli were chosen in line with previous research on color preference in infants (Jadva et al., 2010). Lighting conditions could not be standardized across field sites, so the colors may have appeared slightly different to different participants, due to differences in viewing conditions.

Three pairs of stimuli were colored squares: pink square/blue square, pink square/pale blue square, and red square/blue square. The final two pairs of stimuli were colored line drawings of toys: pink doll/blue doll and pink car/blue car. These stimuli were adapted from a larger measure of color and shape preference (Jadva et al., 2010). The final set of stimuli are shown in Figure S1. The order of presentation was counterbalanced so that the red/pink hues did not always appear on the same side of the page. Scores were calculated as one point per pink/red choice so that children could have a final pink preference score between 0 and 5.

## 3 Results

### 3.1 Effects of Sex on Preference for Pink

**Figure 2:**
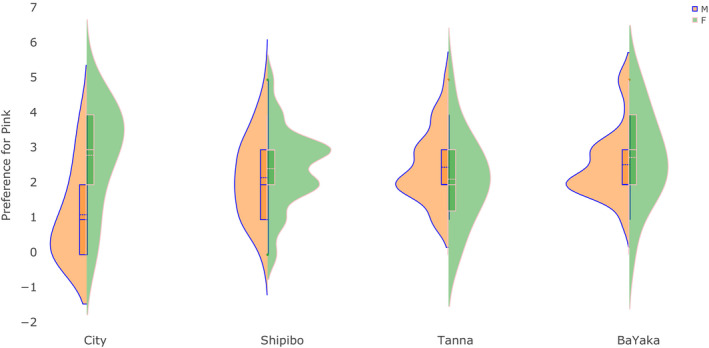
Distribution of boys’ and girls’ preference for pink in each cultural group. Scores are the number of times children chose an option colored pink or red. Within each violin is a boxplot to show sample values, and a density plot to show estimates for the larger population from which the sample was drawn. Boxplots show difference in sample means between boys and girls in each cultural group. Density plots show the probability density of the sample data at different values. Wider sections of the plot show a higher estimated probability that members of the population will take on the given value shown on the *y*‐axis.

Since neither essentialist nor non‐essentialist theories make specific predictions about children’s preferences for pink and blue in small‐scale societies, we consider the analyses presented here to represent a relatively exploratory versus confirmatory effort. A linear regression with preference for pink as the outcome and participant group, sex, and their interactions as predictors revealed significant interactions of sex and culture (see Table 3). To explore the significant interactions, we conducted separate two‐sample *t* tests to compare boys’ and girls’ preference for pink in each participant group. The City participant group showed a different pattern of color preference to the three small‐scale societies, as shown in Figure 2. There was no significant difference between boys’ and girls’ preference for pink in any of the small‐scale societies with no access to global culture: not in the Shipibo villages in Lake Imiria, *t*(92.24) = 1.14, *p* = .255, the Tanna kastom villages, *t*(32.30) = −1.00, *p* = .325, or the BaYaka forager groups in the Congo basin, *t*(28.06) = 0.57, *p* = .573. We found the expected large sex difference in preference for pink in the City sample such that girls preferred pink more than boys did, *t*(35.58) = 4.09, *p* < .001.

**Table 3:** Participant Group and Sex Effects on Preference for Pink (Range = 0–5)

	Estimate	*SE*	*p*
Sex (0 = female)	−1.63	.34	< .001
Group: BaYaka (0 = city)	−0.12	.38	.750
Group: Shipibo (0 = city)	−0.44	.31	.158
Group: Tanna (0 = city)	−0.74	.38	.050
Sex × Group: BaYaka	1.43	.48	.003
Sex × Group: Shipibo	1.37	.41	.001
Sex × Group: Tanna	1.99	.48	< .001

To explore whether sex differences relate to the specific combination of hue and lightness that is pink or to reddish hues more broadly, a follow‐up analysis explored whether the pattern of results for all stimuli was also seen for all color pairs. A logistic regression on the dark pair (red vs. blue) revealed no significant main effects or interactions of sex or participant group on children’s preference for red over blue. A logistic regression on the light pair (pink vs. pale blue) revealed no significant main effects or interactions of sex or participant group on children’s preference for pink over pale blue (see Table S1 for full results), although children generally preferred pale blue over pink (*p* = .008). Finally, a logistic regression on the (light) pink and (dark) blue square pair revealed the expected sex difference, with girls choosing pink more than boys did, *p* = .031, and follow‐up analyses replicated the initial results: no significant sex differences in the Shipibo villages in Lake Imiria, *p* > .999, the Tanna kastom villages, *p* = .825, or the BaYaka forager groups in the Congo basin, *p* = .073, but the expected sex difference in preference for pink in the City sample such that girls chose pink more than boys did, *p* = .031 (see Table S2). Sex differences in children’s preference therefore appear to be related to the specific combination of lightness and hue that is pink, and not to reddish hues in general.

To test the possibility that the null results in the small‐scale societies could be due to small samples or measurement variation, we further calculated the effect sizes of girls’ preference for pink compared to boys’ preference for pink (standardized mean difference, *d*) and the post‐hoc statistical power to detect a sex difference in preference for pink of comparable magnitude to that found in the City sample. We found negligible effect sizes and high statistical power in the Shipibo villages in Lake Imiria, *d* = 0.23 (95% CI [−0.17, 0.63]), power = 0.99, the Tanna kastom villages, *d* = −0.31 (95% CI [−0.90, 0.29]), power = 0.99, and the BaYaka forager groups in the Congo basin, *d* = 0.18 (95% CI [−0.41, 0.77]), power = 0.99. We found a large effect size in the expected direction in the City sample, *d* = 1.24 (95% CI [0.59, 1.90]), power = 0.99. Together, these results suggested that the null effects were unlikely to be due to low statistical power, and more likely to be due to negligible differences in boys’ and girls’ preference for pink in the small‐scale societies.

### 3.2 Effects of Culture on Preference for Pink

By chance, children might be expected to choose the pink/red option about half of the time. Therefore, one‐sample *t* tests were used to detect whether boys and girls in each culture significantly preferred or avoided pink, more than would be expected by chance (2.5 out of 5 total choices). If the *t*‐value is significant and positive, children are selecting pink more than would be expected by chance, indicating a preference for pink. If the *t‐*value is significant and negative, children are selecting pink less than would be expected by chance, indicating an avoidance of pink.

**Figure 3:**
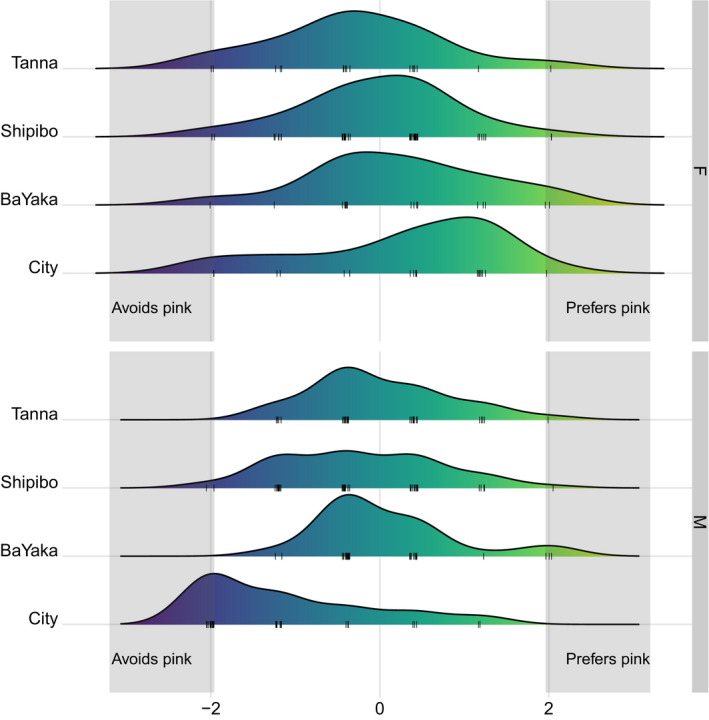
Relative density of preferences for pink in female (above) and male (below) children in the four cultures studied. Scores are the standardized transformation of the number of times children chose a pink/red option. Gray areas indicate significant difference from null (chance) choices, where standardized scores >1.96 indicate significant preference for pink and standardized scores < 1.96 indicate significant avoidance of pink. Lighter colors indicate higher standardized scores and darker colors indicate lower standardized scores.

Results suggested that the sex difference in preference for pink in the City sample was driven by a male avoidance of pink, rather than a female preference for pink (see Figure 3). While girls in no samples showed a significant preference for pink, boys in the City sample showed a significant avoidance of pink, *t*(28) = −5.54, *p* < .001. Table 4 gives the summary statistics for all *t* tests.

**Table 4:** Results for One‐Sample *t* Tests for Preference for, or Avoidance of, Pink. Mean Refers to Mean Number of Pink/Red Choices Out of 5. Probability is Calculated Compared to Chance (Choosing Pink/Red 2.5 Times Out of 5)

Culture	Gender	*M* (*SD*)	*N*	*t*‐value (95% CI)	*p* value	Preference/avoidance
Shipibo	Female	2.46 (1.11)	54	−0.24 (2.16–2.77)	.807	No preference
	Male	2.20 (1.16)	45	−1.74 (1.85–2.55)	.090	No preference
Tanna	Female	2.15 (1.26)	19	−1.18 (1.55–2.76)	.251	No preference
	Male	2.50 (1.01)	30	0.00 (2.12–2.88)	> .999	No preference
BaYaka	Female	2.78 (1.31)	18	0.90 (2.13–3.43)	.380	No preference
	Male	2.58 (1.00)	33	0.43 (2.22–2.93)	.667	No preference
City	Female	2.84 (1.46)	19	1.02 (2.14–3.55)	.322	No preference
	Male	1.14 (1.30)	28	−5.54 (0.64–1.65)	< .001	Avoidance

A follow‐up analysis explored the possible effects of age. Age‐related differences were observed in the City sample, where older boys were less likely to choose pink than younger boys, *b* = −0.33, *SE* = .13, *t* = −2.62, *p* = .015. Children in the small‐scale societies showed no age‐related differences in preference for pink (see Table 5).

**Table 5:** Results for Age‐Related Gender Differences in Preference for Pink in Each Age Group. Each Analysis is a Linear Regression With Age as Predictor and Preference for Pink as the Dependent Variable (Range: 0–5). *N* Refers to the Number of Children in Each Location‐Gender Group Who Had Age Estimates Available

Culture	Gender	Estimate	*SE*	*N*	*p* value
Shipibo	Female	−0.04	.08	52	.637
	Male	0.07	.10	43	.478
Tanna	Female	−0.12	.16	17	.446
	Male	−0.18	.09	28	.063
BaYaka	Female	−0.08	.12	16	.508
	Male	0.06	.08	31	.518
City	Female	−0.29	.22	17	.197
	Male	−0.33	.13	26	.015

## 4 Discussion

We found no significant differences between boys’ and girls’ preference for pink in three small‐scale societies in Peru, Vanuatu, and the northern Congo. We found that girls liked pink more than boys did in a global city, confirming earlier research (Jonauskaite et al., 2019; Mohebbi, 2014; Weisgram et al., 2014; Yeung & Wong, 2018). These results support theories that link color preferences to individual experience (Palmer & Schloss, 2010) and gender cognitions (Bem, 1981; Carter & Levy, 1988; Liben & Bigler, 2002; Martin & Halverson, 1981). That is, culture, not inherent biological dispositions, influences the gender difference in children’s preference for pink.

Our findings contradict essentialist positions that pink is linked to female gender through neural color processing or through evolved preferences linked to foraging or mate choices (Alexander, 2003; Ellis & Ficek, 2001; Hurlbert & Owen, 2015). Supporting our findings, other research indicates that children are not born with sex differences in their color preferences, and that infants show no sex differences in preference for pink until they reach at least 2.5 years of age (Franklin, Gibbons, Chittenden, Alvarez, & Taylor, 2012; Jadva et al., 2010; LoBue & DeLoache, 2011; Wong & Hines, 2015; Zemach et al., 2007). Additionally, some studies of adults in societies with limited access to global culture have found no female preference for pink (Groyecka et al., 2019; Sorokowski et al., 2014), although, as noted before, the female preference for pink over blue may be characteristic of children, rather than adults. Thus, our findings provide additional evidence that the pairing of female and pink is a cultural phenomenon and is not innate.

Results suggest that color preferences are the behavioral expression of a complex interaction between underlying biology and cultural context. Genetic, hormonal, and neural indications may predispose children to display gendered behaviors and preferences, such as color preferences (Arnold, 2009; De Vries & Simerly, 2002; Hines, 2010), but the specific expression of these preferences, such as a female preference for pink, may be learned from cultural setting and individual experience (Bandura, 2002; Carter & Levy, 1988; Martin & Ruble, 2004; Palmer & Schloss, 2010). Children in all cultures are exposed to gender role information that influences their preferences and behavior, but not all cultures include information about the color pink. In our study, male and female roles were well defined and separate in the Vanuatu *kastom* culture (Douglas, 2002; Lindstrom, 2008), while BaYaka (Lewis, 2017) and Shipibo (Hern, 1992) villages were traditionally egalitarian for men and women, although still with typical male and female activities (Ember & Ember, 2003). However, pink was not used in these societies as a marker for female gender. In contrast, in many industrialized settings, boys and girls grow up surrounded by gender color‐coding in marketing, toys, clothing, room decorations, and online (Auster & Mansbach, 2012; Black, Tomlinson, & Korobkova, 2016; Cunningham & Macrae, 2011; Koller, 2008; LoBue & DeLoache, 2011; Pomerleau et al., 1990; Weisgram et al., 2014). Social and cognitive theories would predict that children absorb and integrate this gender color‐coding with a wealth of other gender role information that influences them to show gender differences in color preferences. Indeed, our results suggest that it is cultural norms that influence children’s adoption of gendered preferences and behaviors, such as a female preference for pink.

The specific patterns of color preference seen in our study further suggest that global culture, as well as influencing girls to prefer pink, may influence boys to avoid it. We found that in three small‐scale societies, boys and girls were equally likely to choose a pink option over a blue one. But we found that, like boys in other large industrialized cities (Chiu et al., 2006; Jonauskaite et al., 2019; Mohebbi, 2014; Weisgram et al., 2014; Zentner, 2001), in a large Australian city, boys avoided pink options. This finding supports previous reports that children avoid culturally defined opposite‐sex behaviors (Golombok et al., 2008; Ruble, Martin, & Berenbaum, 2007). Previous research additionally finds that boys increasingly avoid pink choices with age (LoBue & DeLoache, 2011; Wong & Hines, 2015), and this pattern appeared in the boys from our City sample but not in any small‐scale samples, supporting the view that culture may influence boys to avoid girl‐type activities in general and pink specifically. Thus, our findings, in combination with previous research, suggest that the pairing of pink with female gender in global culture might influence boys to avoid options that are colored pink.

It is important to address the cultural bias of color‐coding items for boys and girls. Multiple researchers have suggested that gender‐coding toys by color may affect child development (Martin & Halverson, 1981; Weisgram et al., 2014; Wong & Hines, 2015; Yeung & Wong, 2018). For example, differences in boys’ and girls’ play with toys, that are usually color coded, have been hypothesized to cause sex differences in adult social and spatial skills (Auster & Mansbach, 2012; Martin & Halverson, 1981; Pomerleau et al., 1990). Additionally, cross‐cultural research suggests that sex differences in adult social and spatial skills may also relate to culture (Henrich, Heine, & Norenzayan, 2010; Henrich et al., 2012; Trumble, Gaulin, Dunbar, Kaplan, & Gurven, 2016; Vashro & Cashdan, 2015). Together, this evidence suggests that color‐coding items for boys and girls are not only unnecessary, but may be constraining, as children use these cues to signal what they may be interested in, and what they may want to avoid.

Our study combined children’s responses to red and pink. This choice followed essentialist research that tends to group red with pink as “reddish hues” when explaining sex differences in color preference (Hurlbert & Owen, 2015). Yet, as described in non‐essentialist research (Javda et al., 2010), toys marketed to boys tend to be blue and red, and those marketed to girls tend to be pink, so there may be a cultural reason to consider pink separately from more general “reddish hues.” Our study’s results indicated that sex differences are likely related to the specific color pink, and not to reddish hues in general. Although essentialist viewpoints tend to group pink with red according to hue, our results suggest instead that pink is a separate color that functions as a cultural marker for female gender.

This research investigated children’s preference for pink in small‐scale societies with limited access to global culture via mass media, mass communication, and mass‐produced children’s toys. Results suggested that the pairing of female and pink is a cultural phenomenon and is not driven by an essential preference for pink in girls. Instead, children showed a diversity of preferences with culture. This diversity points to the complex flexibility of underlying biology to drive the development of sex‐typed color preferences in non‐essential, context‐appropriate ways.

## Supporting information

**Figure S1.** Stimuli Used to Test Children’s Preference for Pink
**Table S1.** Gender Differences in Preference for Pink and Red Hues
**Table S2.** Follow‐Up Tests for Gender Differences in Preference for Pink Square Compared to Blue Square
