# Supplementary material for: Cultural Components of Sex Differences in Color Preference
Source: Child Dev. 2021 Jan 21;92(4):1574–89. doi: 10.1111/cdev.13528 (PMC8451877; doi:10.1111/cdev.13528)
Supplement: Supplementary file 1 — Figure S1. Stimuli Used to Test Children’s Preference for Pink Table S1. Gender Differences in Preference for Pink and Red Hues Table S2. Follow‐Up Tests for Gender Differences in Preference for Pink Square Compared to Blue Square [file CDEV-92-1574-s001.docx]

**Supplementary Information**

**Supplementary Figures and Tables**


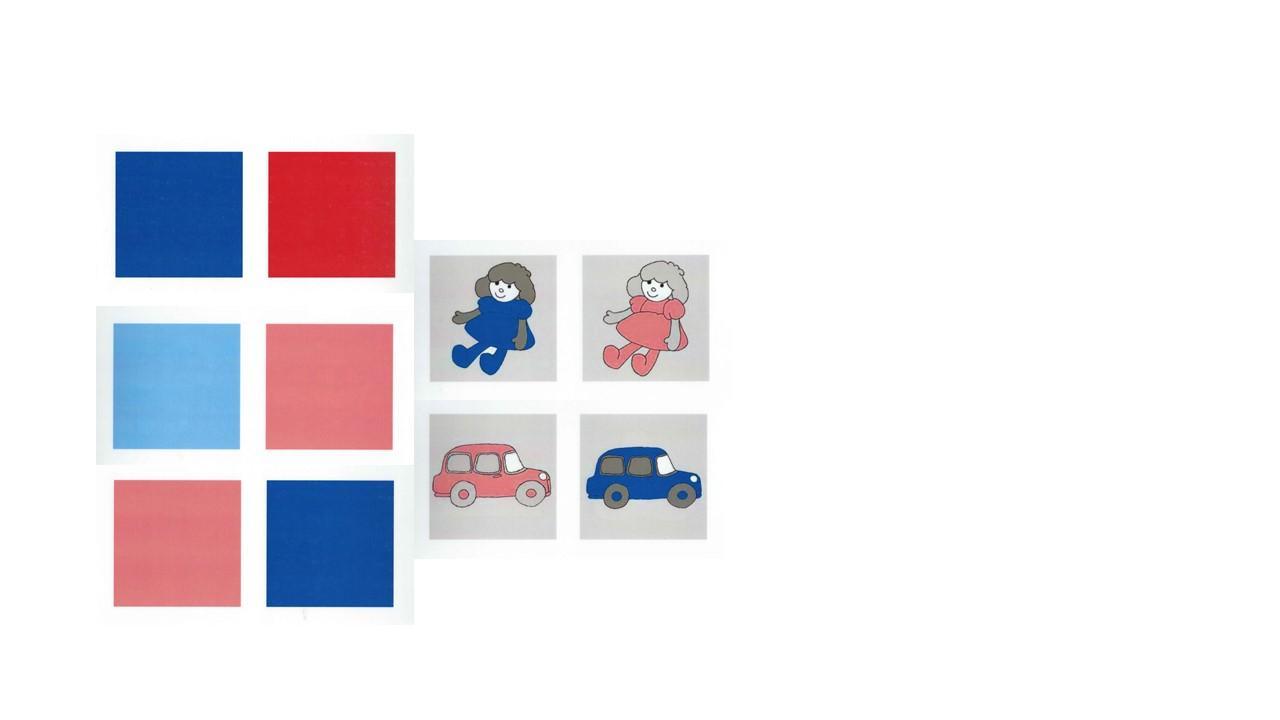


**Figure S1.** Stimuli used to test children’s preference for pink.

**Table S1.** Gender differences in preference for pink and red hues. Each analysis is a logistic regression with sex and participant group as predictors and binary color choice as the dependent variable (0 = pale blue or blue, 1 = pink or red).

| Pair |  | Estimate | SE | *p* |
| --- | --- | --- | --- | --- |
| Red vs. Blue  (0 = Blue) | Intercept | 0.32 | 0.46 | .493 |
|  | Sex  (0 = Female) | -0.75 | 0.60 | .212 |
|  | Group: BaYaka  (0 = City) | -0.10 | 0.66 | .886 |
|  | Group: Shipibo  (0 = City) | -0.10 | 0.54 | .860 |
|  | Group: Tanna  (0 = City) | 0.45 | 0.68 | .502 |
|  | Sex*Group: BaYaka | 0.96 | 0.85 | .256 |
|  | Sex*Group: Shipibo | 0.40 | 0.73 | .585 |
|  | Sex*Group: Tanna | 1.85 | 0.95 | .051 |
| Pink vs. Pale Blue  (0 = Pale Blue) | Intercept | -1.67 | 0.63 | .008 |
|  | Sex  (0 = Female) | 0.15 | 0.80 | .853 |
|  | Group: BaYaka  (0 = City) | 1.45 | 0.79 | .066 |
|  | Group: Shipibo  (0 = City) | 0.98 | 0.69 | .157 |
|  | Group: Tanna  (0 = City) | 1.14 | 0.79 | .150 |
|  | Sex*Group: BaYaka | -0.11 | 0.99 | .914 |
|  | Sex*Group: Shipibo | -0.58 | 0.92 | .525 |
|  | Sex*Group: Tanna | -0.30 | 1.01 | .764 |
| Pink vs. Blue  (0 = Blue) | Intercept | 0.77 | 0.49 | .117 |
|  | Sex  (0 = Female) | -1.36 | 0.63 | .031 |
|  | Group: BaYaka  (0 = City) | 0.48 | 0.75 | .524 |
|  | Group: Shipibo  (0 = City) | -0.55 | 0.56 | .330 |
|  | Group: Tanna  (0 = City) | -1.31 | 0.69 | .056 |
|  | Sex*Group: BaYaka | 0.17 | 0.92 | .854 |
|  | Sex*Group: Shipibo | 1.36 | 0.75 | .070 |
|  | Sex*Group: Tanna | 1.49 | 0.87 | .087 |

**Table S2.** Follow-up tests for gender differences in preference for pink square compared to blue square. Each analysis is a logistic regression with sex as predictor and binary preference for pink as the dependent variable (0 = blue, 1 = pink).

| Participant group |  | Estimate | SE | *p* |
| --- | --- | --- | --- | --- |
| Shipibo | Sex  (0 = Female) | <0.01 | 0.41 | .999 |
| Tanna | Sex  (0 = Female) | -0.13 | 0.60 | .825 |
| BaYaka | Sex  (0 = Female) | -1.19 | 0.67 | .073 |
| City | Sex  (0 = Female) | -1.36 | 0.63 | .031 |
